# A Practical Essay on the Diseases Generally Known under the Denomination of Delirium Tremens

**Published:** 1840-10

**Authors:** 


Art. VII.—*A practical Essay on the Disease generally known under the denomination of Delirium Tremens; written principally with a view to elucidate its division into distinct stages, and to simplify its method of cure.* By Andrew Blake, m.d., Physician to the Nottingham Lunatic Asylum, &c. Second edition, revised and much enlarged. —*London*, 1840. 8vo, pp. 112.

We entered so fully into the subject of this work in our Twelfth Number, (Vol. VI., p. 320,) that we deem it unnecessary to notice at any length the excellent Essay of Dr. Blake. It is but justice to him, however, to state that his work, in its present much improved form, is by far the best monograph we possess on the subject, and well deserves the attention of every practitioner whose personal experience has not prepared him to treat with confidence this dangerous malady. In Dr. Blake he will find an experienced and faithful guide, whom he may safely follow, although he may find the path occasionally a little obscure and circuitous.

